# Empathy and Depression Among Early Adolescents: The Moderating Role of Parental Support

**DOI:** 10.3389/fpsyg.2019.01447

**Published:** 2019-06-28

**Authors:** Emanuela Calandri, Federica Graziano, Silvia Testa, Elena Cattelino, Tatiana Begotti

**Affiliations:** ^1^Department of Psychology, University of Turin, Turin, Italy; ^2^Department of Human and Social Sciences, University of Aosta Valley, Aosta, Italy

**Keywords:** depression, empathy, maternal support, paternal support, early adolescence

## Abstract

Early adolescence is a period of development of emotional competence, but also of increasing vulnerability for the onset of depressive symptoms. While literature underscored that empathy promotes social relationships and psychological well-being over the life course, the possible role of high empathy levels as a risk factor for depression has been under investigated, especially among early adolescents. Moreover, although parenting practices are known to influence both empathy and depression in adolescence, few studies investigated if parenting moderates the relationship between empathy and depression. Therefore, the aims of the study were: (1) to investigate the relationships between affective and cognitive empathy and depression; (2) to investigate the moderating role of perceived paternal and maternal support on the associations between affective and cognitive empathy and depression; (3) to examine if the relationships among affective and cognitive empathy, maternal and paternal support and depression vary as a function of early adolescents’ gender. The study involved 386 Italian students aged between 12 and 14 (*M* age = 13, *SD* = 0.3, 47.9% girls) who completed an anonymous self-report questionnaire, including measures of cognitive and affective empathy, paternal and maternal support and depression. Results showed that with a mean level of affective and cognitive empathy, higher maternal support was related to lower depression for girls, whereas higher paternal support was related to lower depression for both boys and girls. Both maternal and paternal support moderated the relation between empathy and depression. In particular, maternal support moderated the non-linear relation between affective empathy and depression and the relation was further moderated by early adolescents’ gender: boys with low affective empathy reported lower depression in a context of high maternal support. Paternal support moderated the linear relation between cognitive empathy and depression, independently of early adolescents’ gender: boys and girls with high cognitive empathy reported higher depression in a context of low paternal support. The results of the study suggested that high empathy might be a risk factor for depression during early adolescence and mothers and fathers have a differential moderating role in relation to the affective and cognitive dimensions of empathy, also in relation to early adolescents’ gender.

## Introduction

Early adolescence is a period of great developmental challenges: the pubertal transition is associated with many physical and psychological changes, that can be linked with the increase of emotional and social competence, but also with the increasing vulnerability for depressive symptoms, especially for girls (Hamilton et al., 2014). Literature has repeatedly stressed that rates of depressive symptoms start to increase from early adolescence onwards (Rudolph, 2002; Olino et al., 2014; McLaughlin and King, 2015). Also gender differences in depression begin to emerge during early adolescence (around 12–13 years of age) and become more pronounced across adolescence, with girls twice as likely to be depressed as boys (Hankin et al., 2007; Avenevoli et al., 2008). While most studies investigated risk and protective factors of depression in middle and late adolescence (see Cairns et al., 2014 for a review), there is a lack of knowledge on correlates of depressive symptoms in early adolescence. Identifying these factors is relevant to implement timely interventions to contrast depression and promote positive developmental trajectories among early adolescents.

### Empathy and Depression

Empathy has been defined as an emotional response to the affective state or situation of other people and it is considered a multidimensional construct, including the ability to recognize and understand another’s feelings (cognitive dimension) and to share and vicariously experience those emotions (affective dimension) (Feshbach, 1997; Hoffman, 2008). Empathy emerges in the early childhood and become more complex during the individual development (Eisenberg et al., 2013). Early adolescence is a particularly critical period for empathy development (van Lissa et al., 2014). The great number of physical and psychological changes, the improvement of abstract thinking and the changes in moral reasoning, combined with individual and social transitions, constitute significant challenges that have important implications for empathy development (Allemand et al., 2015).

Many studies have stressed the positive role of empathy in increasing interpersonal and mental health outcomes (Chow et al., 2013). Affective and cognitive empathy are related to adolescents’ interpersonal functioning, promoting prosocial behavior (Van der Graaff et al., 2018) and inhibiting aggressive and externalizing problem behaviors (Laible et al., 2004). Empathy results as an adaptive characteristic especially when both cognitive and affective dimensions are moderate and well regulated; this type of empathy is related with the greatest social benefits, because it allows to understand others’ emotions and to get affectively involved without becoming overwhelmed (Tully et al., 2016). Low empathy is instead associated with more conflicts, aggressive behaviors and bullying (Jolliffe and Farrington, 2004; Gini et al., 2007). Individuals with low empathy cannot imagine the consequences of their behavior and the potential harm they might cause. Recent research has also highlighted the critical role of high levels of empathy among adolescents and adults. Some studies found that extreme sympathy and compassion, as a response to other people’s suffering, may lead to consequent prolonged and exhausting empathic reactions (Smith and Rose, 2011; Oakley et al., 2012). This situation of personal distress is related to withdrawal, avoidance of empathy-inducing situations, and depression (Schreiter et al., 2013). In particular, high empathy may increase the risk for depression, if associated with particular individual and contextual characteristics which act as moderators or mediating factors (Hoffman, 2000; Hodges and Biswas-Diener, 2007; O’Connor et al., 2007; Tone and Tully, 2014). Among individual factors, gender is significantly related to empathy; not only girls generally report higher empathy, especially affective, than boys (Allemand et al., 2015), but high levels of affective empathy have a stronger and more significant association with internalizing symptoms in girls than in boys (Bell et al., 2005; Gambin and Sharp, 2016). Contextual variables also have an effect on the relationship between empathy and adjustment problems in adolescence. In particular, recent studies examined the role of family environment variables, in particular parenting practices (Tone and Tully, 2014; Green et al., 2018) and conflict with parents (Van Lissa et al., 2017) as moderating factors in the relation between empathy and depression. The relationship between empathy and depression is therefore complex and there is a need to deepen knowledge about variables that act as moderators, especially among early adolescents.

### Maternal and Paternal Support and Depression

During early adolescence, the changes in family relationships can play a significant role in the improvement of adjustment problems. Contemporary studies have stressed that during early adolescence, girls and boys are surprisingly resilient in facing the normative challenges typical of the period, especially if they can count on the support of some caring adults (Steinberg, 2001; Tuggle et al., 2014). Among family factors, a central role is played by parental support, defined as the amount of acceptance or warmth that parents express to their children (Bean et al., 2006; Adams and Laursen, 2007). Supportive parents play a decisive role in promoting the healthy adjustment of their adolescent offspring (Rueger et al., 2010). While low levels of parental support may increase psychological distress and emotional problems (Demaray et al., 2005), high levels of parental support may promote those beliefs such as acceptance, self-esteem, trust and confidence in others, that are negatively associated with depressive feelings (Helsen et al., 2000; Colarossi and Eccles, 2003; Newman et al., 2007).

Most studies aggregated maternal and paternal support into a unique measure; therefore, there is a need of investigating the specific contribution of mothers’ and fathers’ support on adolescents’ adjustment (Di Maggio and Zappulla, 2014). While maternal support is acknowledged to be a protective factor against adolescents’ depression (Vaughan et al., 2010), recent literature has given greater attention also on the role of fathers in adolescents’ development and adjustment (Day and Padilla-Walker, 2009; Graziano et al., 2009; Babore et al., 2016). In particular, a good quality of father involvement and support can have a positive influence on adolescents’ management of stressful or sad situations, may improve adolescents’ life satisfaction and decrease the risk of depression (Antonopoulou et al., 2012). Indeed, maternal and paternal support may vary in terms of quantity and quality and may be functionally different on the base of individual adolescents’ characteristics, such as their gender (Moilanen et al., 2015). As stressed by Colarossi and Eccles (2003), to better understand the effects of family support on adolescents’ healthy adjustment it is essential to analyze the interactions between the gender of the support provider (for example, mother vs. father) and the gender of the recipient (girl vs. boy adolescents). Findings of Colarossi and Eccles (2003) indicated that boys perceived significantly more support from fathers than girls, while no gender differences were found in perception of support from mothers, who were perceived as higher supportive than fathers both by boys and girls. Moreover, mother support was negatively associated to depressive feelings especially in girls, while father support had larger effects on boys’ depression. These results suggest that the effect of parental support may be particularly strong in a same-sex couple (mother-daughter or father-son).

### Maternal and Paternal Support as Moderators of the Relationship Between Empathy and Depression

In light of the examined literature, the association between parent-adolescent relationships and early adolescents’ adaptive emotional socialization appears to be very complex. Family variables can influence early adolescents’ adjustment not only directly, but also through moderation and mediation effects. As suggested by Tone and Tully (2014), excessive empathy in combination with maladaptive parenting might be linked to increased risk for internalizing problems. Nonetheless, only a little research examined this topic. Zahn-Waxler and Van Hulle (2012) postulated that children and adolescents with high empathy might develop pathogenic guilt when parents are unsupportive and excessively demanding, and this can result in an increased likelihood of depression. Other studies specifically examined the moderating or the mediating role of maternal support on the relationship between empathy and depression, whereas the role of paternal support remains largely unexplored. In particular, Green et al. (2018) demonstrated that in the context of a negative mother–adolescent relationship, high affective empathy acts as a risk factor for depressive symptoms among adolescents. Finally, Soenens et al. (2007) examined maternal support in relation to adolescents’ empathy dimensions, in particular maternal support as mediator of the intergenerational similarity between mothers’ and adolescents’ empathy-related responding. They found that maternal support mediates the relation between maternal and adolescent cognitive empathy, thus suggesting that cognitive empathy is transmitted from mothers to adolescents through maternal supportive rearing style. To our knowledge, no studies have yet considered the moderating role of both maternal and paternal support in the relation between empathy and depression among early adolescents.

### The Present Study

The present study expanded the existing literature on the association between empathy and depression and on the moderating role of maternal and paternal support during early adolescence. In particular, the aims were:

(1) to describe levels of depression, affective and cognitive empathy, paternal and maternal support in a group of early adolescents, taking into account gender differences. Consistently with results of previous literature, we expected that girls report higher depression (Olino et al., 2014; McLaughlin and King, 2015), higher empathy (Allemand et al., 2015), and lower paternal support (Colarossi and Eccles, 2003) than boys.
(2) to investigate the relationships between empathy (both affective and cognitive) and depression; moving from studies that indicated the adaptive role of moderate levels of empathy with respect to adjustment (Tully et al., 2016), we expected that both extremely high and extremely low affective and cognitive empathy would be associated with higher depression. The role of extreme levels of empathy was examined by considering the quadratic associations between empathy (both affective and cognitive) and depression.
(3) to investigate the potential moderating role of paternal and maternal support, as perceived by early adolescents, on the associations between empathy (both affective and cognitive) and depression; both maternal and paternal support were expected to be linked to lower depression (Vaughan et al., 2010; Antonopoulou et al., 2012). Moreover, we expected that both maternal and paternal support moderated the associations between affective and cognitive empathy and depression (Tone and Tully, 2014; Green et al., 2018). In particular, we expected that the association between extreme levels of empathy and depression was weaker when perceived parental support was high. As for differences between paternal and maternal support, the study was explorative in nature and no specific hypothesis was formulated.
(4) to examine if the relationships among empathy (affective and cognitive), maternal and paternal support and depression vary as a function of early adolescents’ gender. In particular, we investigated if the gender of early adolescents moderates: (a) the relationship between empathy and depression (two-way interactions); (b) the relationship between paternal/maternal support and depression (two-way interactions); (c) the relationship between empathy and depression as moderated by paternal/maternal support (three-way interactions); all these analyses were exploratory in nature and no specific hypotheses were formulated.

## Materials and Methods

### Participants

A convenience sample of 7 middle schools located in urban centers in the North-West of Italy was selected to participate in the study. The research project was presented to each school and a total of 26 s year^1^ classes were enrolled. The sample was composed of 386 early adolescents aged between 12 and 14 (*M* age = 13, *SD* = 0.3) (*N* = 185, 47.9% girls). The majority of participants (*N* = 328, 85.2%) lived with both parents and had brothers or sisters (*N* = 329, 85.2%). Parents’ level of education was medium-high (high school diploma for 25.3% of mothers and 20.4% of fathers; degree for 25.3% of mothers and 19% of fathers). The majority of parents were employed full time (56.1% of mothers and 83.8% of fathers).^2^

### Procedure

Participants completed an anonymous self-report questionnaire, administered by trained researchers in the schools during classroom time, without teachers present. Completed questionnaires were turned in immediately to researchers. Participants did not receive benefits for participating in the study. The study was approved by the Bioethics Committee of the University of Turin (Italy) and written informed consent was obtained from the parents of the participants before the questionnaire was administered. Parental consent was given for 96% of the students originally contacted to participate in the study.

### Measures

#### Empathy

Students were asked to complete the scale How I feel in different situations (HIFDS, Feschbach et al., 1991, Italian validation Bonino et al., 1998). It is composed of 12 items investigating cognitive empathy (6 items) (e.g., “I can sense how my friends feel from the way they behave”) and affective empathy (6 items) (e.g., “When somebody tells me a nice story, I feel as if the story is happening to me”) on a 4-point Likert scale from 0 (never true) to 3 (always true) (the score of each scale ranged from 0 to 18). In this study, Cronbach’s alfas were 0.79 for affective empathy and 0.76 for cognitive empathy, respectively.

#### Paternal and Maternal Support

Students completed the scale of parental support formulated by Kerr et al. (2010). It is composed of 10 items evaluating the perceived support, closeness, help and encouragement from mothers (5 items) and fathers (5 items) (e.g., “When I am angry, sad or worried, my mother/father can make me feel better”). The agreement is expressed on a 4-point Likert scale from 0 (not agree) to 3 (agree very much) (the score of each scale ranged from 0 to 15). In this study, Cronbach’s alfas were 0.82 for maternal support and 0.87 for paternal support, respectively.

#### Depression

Students completed the CESD-10 (Center for Epidemiological Studies Scale- short version 10 items) in the Italian validation (Pierfederici et al., 1982). The scale evaluates the frequency of depressive symptoms during the past week on a 4-point Likert scale from 0 (rarely or none of the time) to 3 (most or all of the time) (range 0–30, Cronbach’s alfa = 0.69). A cut-off score of 10 indicates the presence of clinically significant depressive symptoms.

### Statistical Analysis

A preliminary check on missing data indicated that the percentage of missing response for the study scales was less than 10%. The MCAR (Missing Completely at Random) test (Little, 1988) showed non-significant results for affective empathy, cognitive empathy and paternal support, thus missing were imputed in SPSS with the EM (Expectation-Maximization) procedure. Since the MCAR test showed significant results for depression and maternal support, indicating that missing were not completely at random, the imputation was carried out through the Regression procedure. Preliminary descriptive analyses included *t*-tests for gender differences in study variables, Cohen’s d as a measure of *t*-test effect size, and Pearson’s bivariate correlations. Then, according to the aims of the study, we ran two regression models to predict depression. In the first model, the focal predictor was affective empathy (both linear and quadratic) and gender, maternal and paternal support were moderator variables, whereas in the second model the focal predictor was cognitive empathy (both linear and quadratic) and gender, maternal and paternal support were, as before, the moderator variables. Both models included main effects, as well as all two-way and three-way interactions. The focal independent variables (affective and cognitive empathy) and maternal and paternal support were mean centered prior to analyses (Aiken and West, 1991), whereas gender was dummy coded (1 = boy). To interpret significant interactions with linear variables, we plotted the effects and performed a simple slope analysis. For moderating continuous variables (maternal and paternal support) we tested the effects at low (mean −1 sd) and high (mean +1 sd) levels of the moderator.

Following the approach suggested by Dawson (2014), a significant interaction that included a quadratic term was examined only if the increase of explained variance obtained after the introduction of this term and its linear counterpart was statistically significant. Then the interaction effect was plotted and both simple slope and slope difference tests were performed. The indirect method was followed: the moderators were centered around low and high values and the regression model was re-run using these new variables in turn (Dawson, 2014). All statistical analyses were performed with SPSS Statistics 25.

## Results

### Descriptive Analysis

Means and standard deviations of study variables are reported in Table 1. As for depression, the majority of participants (*N* = 300, 77.7%) had a score lower than 10, which represent the critical cut-off for the presence of clinically significant depressive symptoms; 68 early adolescents (17.6%) reported a score ranging from 10 to 15 and the remaining (*N* = 18, 4.7%) had a score higher than 15. Depression scores were higher for girls than for boys. Girls also reported higher affective empathy than boys, whereas boys reported higher paternal support than girls (Table 2). Bivariate correlations among study variables indicated that depression was positively related to affective empathy and negatively to paternal and maternal support. The two components of empathy were positively correlated. Affective empathy was positively related to maternal support, whereas cognitive empathy was positively related to both maternal and paternal support. Finally, maternal and paternal support were positively interrelated (Table 1).

**TABLE 1:** Descriptive statistics and bivariate correlations among study variables.

	***M* (*SD*)**	**1**	**2**	**3**	**4**	**5**	**6**	**7**
1. Depression	7.2⁢(4.1)	1						
2. Linear AE	7.6⁢(3.7)	0.12^*^	1					
3. Quadratic AE	–	0.02	0.21^∗∗^	1				
4. Linear CE	9.8⁢(3.7)	0.05	0.48^∗∗^	0.05	1			
5. Quadratic CE	–	–0.05	–0.04	0.33^∗∗^	–0.09	1		
6. M_SUP	11.5⁢(3.3)	–0.26^∗∗^	0.17^∗∗^	–0.08	0.16^∗∗^	–0.02	1	
7. P_SUP	10.8⁢(3.8)	–0.34^∗∗^	0.02	–0.02	0.11^*^	0.07	0.48^∗∗^	1

AE, Affective Empathy; CE, Cognitive Empathy; M_SUP, Maternal Support; P_SUP, Paternal Support. ^*^p < 0.05, *^∗∗^p < 0.01.*

**TABLE 2:** Gender differences in study variables.

	**Girls**		**Boys**		**Student’s t_(df)_**	***p***	**Cohen’s d**
	***M***	***SD***	***M***	***SD***			
Depression	8.16	4.35	6.35	3.67	$-4.39{}_{\left(384\right)}$	0.0001	0.45
Affective empathy	8.72	3.57	6.48	3.56	$-6.16{}_{\left(384\right)}$	0.0001	0.63
Cognitive empathy	10.13	3.57	9.51	3.77	$-1.66{}_{\left(384\right)}$	0.098	0.17
Maternal support	11.43	3.28	11.57	3.39	$0.41{}_{\left(384\right)}$	0.681	0.04
Paternal support	10.06	3.97	11.39	3.53	$3.49{}_{\left(384\right)}$	0.0001	0.36

### Affective Empathy and Depression: Moderation Analysis

**FIGURE 1:**
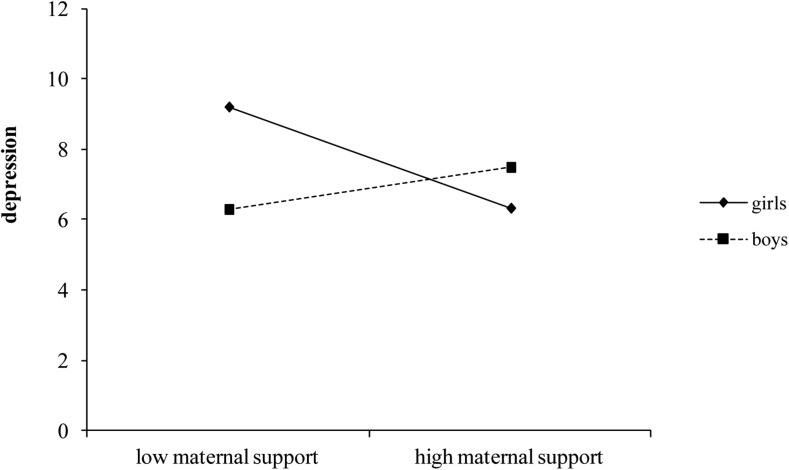
Moderatingeffect of gender on the relationship between maternal support and depression (controlling for affective empathy).

Results of the first regression model are reported in Table 3. The model explained 20% of the variance in depression scores. Significant coefficients were observed for maternal support, paternal support, as well as for the two-way interaction maternal support X gender, and for the three-way interaction quadratic affective empathy X maternal support X gender. The increase in the explained variance after entering both linear and quadratic terms of the 3-way interaction was statistically significant [Δ*R*^2^ = 0.017; *F*_(2,__368)_ = 3.88, *p* < 0.05]. Paternal support was negatively related to depression, regardless of the gender of the adolescents or their level of affective empathy (none of the interactions involving paternal support was statistically significant). Higher levels of paternal support were associated to lower levels of depression. Regarding maternal support, results indicated that its association with depression was moderated by gender: high levels of maternal support were related to lower depression only for girls. The simple slope was in fact significant for girls (*b* = −0.431, *t* = −3.734, *p* < 0.001), but not for boys (*b* = 0.179, *t* = 1.326, *p* = 0.186; Figure 1).

**TABLE 3:** Multiple regression analysis with moderating effects of maternal support, paternal support, and gender on the relationship between affective empathy (linear and quadratic) and depression.

	***B***	***SE B***	**β**	***p***
Intercept	7.760	0.358		
Gender	–0.876	0.519	–0.107	0.092
Linear AE	0.130	0.097	0.118	0.180
Quadratic AE	0.003	0.019	0.013	0.865
M_SUP	**-0.431**	**0.115**	**-0.351**	**0.0001**
P_SUP	**-0.259**	**0.095**	**-0.240**	**0.007**
Linear AE X gender	–0.037	0.129	–0.023	0.776
Quadratic AE X gender	–0.031	0.028	–0.097	0.270
M_SUP X gender	**0.610**	**0.177**	**0.363**	**0.001**
P_SUP X gender	–0.265	0.159	–0.165	0.096
Linear AE X M_SUP	–0.030	0.034	–0.100	0.372
Linear AE X P_SUP	–0.007	0.028	–0.027	0.792
Quadratic AE X M_SUP	0.011	0.006	0.230	0.066
Quadratic AE X P_SUP	0.003	0.005	0.064	0.549
Linear AE X M_SUP X gender	0.076	0.047	0.193	0.105
Linear AE X P_SUP X gender	–0.016	0.038	–0.039	0.678
Quadratic AE X M_SUP X gender	**-0.020**	**0.009**	**-0.333**	**0.020**
Quadratic AE X P_SUP X gender	0.009	0.008	0.136	0.235

**AE, Affective Empathy; M_SUP, Maternal Support; P_SUP, Paternal Support.Full model R^2^ = 0.20; F_(17, 368)_ = 5.55, p < 0.001; Model with only the linear term of gender, affective empathy, maternal and paternal support variables) R^2^ = 0.16; F_(4, 381)_ = 18.705, p < 0.001; statistically significant betas (*p* < 0.05).**

**FIGURE 2:**
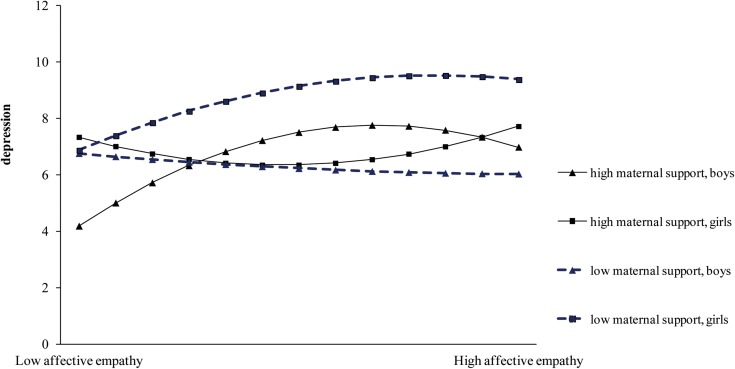
Moderating effect of maternal support and gender on the curvilinear relationship between affective empathy and depression.

The significant three-way interaction between quadratic affective empathy, maternal support and gender indicated that gender moderates the influence of maternal support on the relationship between affective empathy and depression (Figure 2). The simple slope analysis revealed that only the slope of boys with high maternal support was statistically significant [Δ*R*^2^ = 0.018; *F*_(__2,__368)_ = 4.11, *p* = 0.017]. With slope difference analyses, we compared girls with high (mean +1 sd) and low (mean −1 sd) maternal support, as well as boys with high (mean +1 sd) and low (mean +1 sd) maternal support, obtaining a significant difference only between boys with high and low support [Δ*R*^2^ = 0.018; *F*_(2,__368)_ = 4.23, *p* = 0.015]. To summarize, only among boys with high maternal support, affective empathy was related to depression. In particular, boys with a high maternal support and a low affective empathy shown the lowest scores on depression.

### Cognitive Empathy and Depression: Moderation Analysis

Results of the second regression model are reported in Table 4. The model explained 21% of the variance in depression scores. Significant coefficients were observed for gender, cognitive empathy, maternal support, paternal support, as well as for the two-way interactions maternal support X gender, cognitive empathy X paternal support, and quadratic cognitive empathy X maternal support. The increase in the explained variance after entering both linear and quadratic terms of the last 2-way interaction was not statistically significant [Δ*R*^2^ = 0.009; *F*_(2,__368)_ = 2.07, *p* = 0.127], thus this interaction was no further examined.

**TABLE 4:** Multiple regression analysis with moderating effects of maternal support, paternal support, and gender on the relationship between cognitive empathy (linear and quadratic) and depression.

	***B***	***SE B***	**β**	***P***
Intercept	7.926	0.358		
Gender	**-1.304**	**0.497**	**-0.159**	**0.009**
Linear CE	**0.198**	**0.083**	**0.178**	**0.018**
Quadratic CE	0.001	0.018	0.003	0.966
M_SUP	**-0.470**	**0.118**	**-0.382**	**0.0001**
P_SUP	**-0.224**	**0.092**	**-0.207**	**0.015**
Linear CE X gender	–0.126	0.114	–0.084	0.269
Quadratic CE X gender	–0.004	0.025	–0.014	0.870
M_SUP X gender	**0.477**	**0.167**	**0.284**	**0.004**
P_SUP X gender	–0.109	0.145	–0.068	0.452
Linear CE X M_SUP	0.002	0.025	0.007	0.934
Linear CE X P_SUP	**-0.054**	**0.025**	**-0.174**	**0.034**
Quadratic CE X M_SUP	**0.011**	**0.005**	**0.234**	**0.042**
Quadratic CE X P_SUP	–0.004	0.005	–0.083	0.362
Linear CE X M_SUP X gender	0.028	0.040	0.068	0.488
Linear CE X P_SUP X gender	–0.014	0.039	–0.032	0.713
Quadratic CE X M_SUP X gender	–0.012	0.007	–0.215	0.085
Quadratic CE X P_SUP X gender	0.005	0.007	0.064	0.490

**CE, Cognitive Empathy; M_SUP, Maternal Support; P_SUP, Paternal Support.Full model: R^2^ = 0.21; F_(17, 368)_ = 5.89, p < 0.001; Model with only the linear term of gender, cognitive empathy, maternal and paternal support variables) R^2^ = 0.16; F_(4, 381)_ = 18.210, p < 0.001; statistically significant betas (*p* < 0.05).**

**FIGURE 3:**
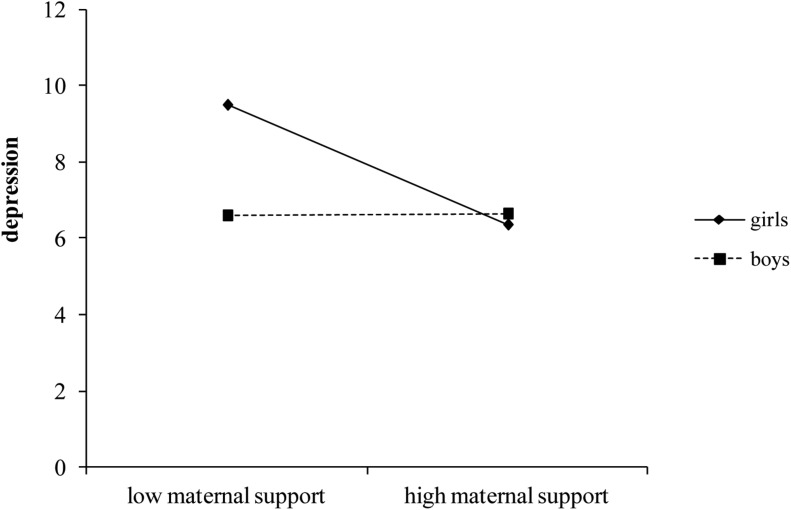
Moderating effect of gender on the relationship between maternal support and depression (controlling for cognitive empathy).

As found in the previous model, high levels of maternal support were related to lower depression only for girls (simple slope for girls, *b* = −0.470, *t* = −3.983, *p* < 0.001; simple slope for boys, *b* = 0.007, *t* = 0.060, *p* = 0.953; Figure 3), and, as before, paternal support was negatively related to depression, regardless of the gender of the adolescents.

**FIGURE 4:**
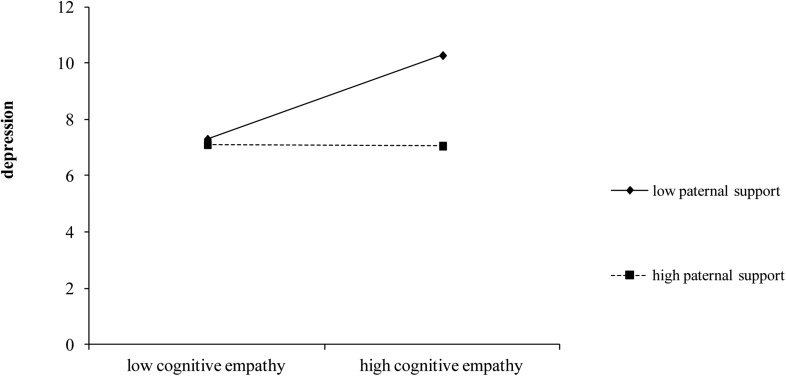
Moderating effect of paternal support on the relationship between cognitive empathy and depression.

Moreover, higher depression was related to being girls and to having high cognitive empathy scores. This last positive association was present both for the average level of paternal support, as indicated by cognitive empathy main effect, and for low level of paternal support, as shown by the simple slope analysis of the interaction between cognitive empathy and paternal support. In fact, high levels of cognitive empathy were related to higher depression in a context of low perceived support from fathers (simple slope for low paternal support, *b* = 0.403, *t* = −3.146, *p* < 0.01) but there was not an association for high paternal support, simple slope: *b* = −0.007, *t* = −0.057, *p* = 0.954; Figure 4).

### Summary of Results

To sum up, the results of the study indicated that:

(1) with a mean level of affective and cognitive empathy, higher maternal support was related to lower depression for girls, whereas higher paternal support was related to lower depression for both boys and girls.
(2) maternal support had a moderating role in the quadratic relation between affective empathy and depression and the relation was further moderated by early adolescents’ gender: boys with low affective empathy shown the lowest scores on depression in a context of high perceived maternal support.
(3) paternal support had a moderating role in the linear relation between cognitive empathy and depression, independently of early adolescents’ gender: high levels of cognitive empathy were related to higher depression when boys and girls perceived lower support from fathers.

## Discussion

The study was aimed at investigating the relationships between empathy (both affective and cognitive) and depression in early adolescence, examining the moderating role of maternal and paternal support and taking into account early adolescents’ gender differences. The study suggested that the association between empathy and depression is complex and parental support plays a central role with some differences between boys and girls.

With regard to the first aim, our descriptive results were consistent with previous studies, indicating higher depression (Olino et al., 2014; McLaughlin and King, 2015) and higher affective empathy (Allemand et al., 2015) among girls. Also in our sample of early adolescents, girls seem therefore more at risk than boys to experience depressive symptoms, and more prone to share and vicariously experience others’ emotions than their male peers. This difference in levels of affective empathy could be explained with reference to cultural models that encourage girls to be sensitive to other’s emotional difficulties and to place more importance to intimacy than boys in peer relationships (Rubin et al., 2006). Moreover, we found that boys reported higher paternal support than girls, consistent with previous literature, suggesting that paternal warmth and closeness are perceived as particularly salient in the son-father couple (Colarossi and Eccles, 2003).

Concerning the second aim, the hypothesis that extreme levels of empathy would be associated with higher depression was partially confirmed, in line with previous studies highlighting the links between excessive empathy and internalizing problems (Oakley et al., 2012; Schreiter et al., 2013; Tully et al., 2016). Even though empathy is acknowledged to be an important life skill and is related to psychological well-being and positive adjustment (Laible et al., 2004; Chow et al., 2013), the present study suggests the potential risk associated with extreme and excessive form of empathic responses, in absence of a moderating role of parenting.

As for the third aim, we found that both maternal and paternal support were protective against depression when associated with a mean level of affective and cognitive empathy. In line with previous studies (Tone and Tully, 2014; Green et al., 2018), we found that both maternal and paternal support moderated the associations between empathy and depression. The hypothesis that the association between extreme levels of empathy and depression would be weaker when perceived parental support was high was partially confirmed.

Interesting results emerged when considering the specific role of paternal and maternal support and early adolescents’ gender as further moderating variable (fourth aim). First of all, the role of maternal support on depression seems to be more influential for girls, whereas the role of fathers seems to be equally important for both boys and girls. On the one hand, this result is consistent with research highlighting the strong effect of parental support in the mother-daughter couple (Colarossi and Eccles, 2003). On the other hand, our findings stressed the central role played by fathers in the relationship with their offspring, especially during early adolescence (Day and Padilla-Walker, 2009; Antonopoulou et al., 2012; Babore et al., 2016). Secondly, maternal and paternal support seem to differentially moderate the two components of empathy (affective and cognitive), in relation to early adolescents’ gender. On the one hand, boys with low levels of affective empathy refer lower depression when they can rely on a high maternal support. On the other hand, boys and girls with high cognitive empathy reported lower depression when they perceived high paternal support. This differential role of maternal and paternal support in relation to the different dimensions of empathy is in line with the study of Miklikowska et al. (2011), where a greater maternal influence on adolescents’ empathic concern and a greater paternal influence on perspective taking were found. These differences might be due to differential parenting styles: while paternal support is more likely to act through a process of cognitive sharing of problems and search for solving strategies, maternal support is more likely to be linked to a process of affective sharing of emotions. Our results are therefore preliminary and the topic deserves further investigation.

The present study expands the existing literature on the complex associations between empathy and depression and the moderating role of maternal and paternal support during early adolescence. The strength of this study lies in considering the nonlinear relation between empathy and depression. This type of analysis allowed to go beyond the role of maternal support when girls report mean levels of empathy and to highlight the importance of maternal support for boys with extremely low levels of affective empathy. Nonetheless, the study had some limitations. First of all, the sample is not representative, thus limiting the generalizability of results. Second, the cross-sectional nature of the research allows to investigate the association between variables, but it limits the possibility to interpret the directionality of the relations. Third, our study only relied on self-report measures and this would have partially biased the study results.

To overcome these limitations, further research should involve a larger and representative sample in order to confirm the preliminary results of the present study. Longitudinal investigation would be useful to investigate the directionality of the relation between empathy and depression and the moderating role of parenting variables. Further longitudinal research would be also useful to study the specific protective role of paternal and maternal support with respect to subsequent depressive symptoms. Finally, further research should integrate self-report measures of parenting with maternal and paternal points of view to clarify the moderation role of parental support on the links between empathy and depression.

Our results have relevant implications for prevention and intervention to contrast depression in early adolescence. Recent research highlighted the need of individuating key variables of children and adolescents depression in order to implement effective programs specifically targeting this population (Bernaras et al., 2019). Empathy and parental support might be two core elements to consider in prevention programs specifically targeting early adolescents. On the one hand, programs of empathy promotion for early adolescents must take into account that empathy is a life skill relevant for positive interpersonal relationships. On the other hand, there must be an awareness that extreme forms of empathy could expose early adolescents, especially girls, to a greater risk of depression, if not associated to the promotion of protective factors in the family context. Community-based interventions should be focused on the promotion of parenting abilities, in particular parental support, that might contrast depressive feelings, especially when early adolescents report extremely high levels of empathy involvement. As for clinical interventions, moving from the consideration that early adolescents with extreme empathy are more likely to develop depressive symptoms when parents are less supportive, clinicians should pay attention to parental support as a key variable of intervention. In particular, a final consideration concerns the role of paternal support with respect to early adolescents’ depression. In light of the results of the present study, it seems appropriate to help fathers to be aware of the importance of being highly supportive toward their offspring to contrast depressive feelings associated with high levels of empathy, especially in the cognitive component.

## Ethics Statement

The study was approved by the Turin University Bioethical Committee (Prot. n. 3175 del 1/2/2016) and parental and personal consent were required before the questionnaire was administered.

## Author Contributions

EMC conceived the study and wrote the manuscript. ST provided statistical analysis and interpretation of the results. FG provided statistical analysis and wrote the manuscript. TB collaborated in writing introduction and discussion of the manuscript and editing of the manuscript. ELC had project supervision and monitored the progress of the study.

## Conflict of Interest Statement

The authors declare that the research was conducted in the absence of any commercial or financial relationships that could be construed as a potential conflict of interest.

## Footnotes

1 In the Italian school system, there is a first cycle of education that lasts 5 years (primary school, from 6 to 11 years of age) and a second cycle that lasts 3 years (middle school, from 11 to 14 years of age).

2 Family structure and parental levels of education were in line with Italian population data (ISTAT, 2016).
